# Hybrid Simulation and Planning Platform for Cryosurgery with Microsoft HoloLens

**DOI:** 10.3390/s21134450

**Published:** 2021-06-29

**Authors:** Sara Condino, Fabrizio Cutolo, Nadia Cattari, Simone Colangeli, Paolo Domenico Parchi, Roberta Piazza, Alfio Damiano Ruinato, Rodolfo Capanna, Vincenzo Ferrari

**Affiliations:** 1 Information Engineering Department, University of Pisa, 56126 Pisa, Italy; fabrizio.cutolo@endocas.unipi.it (F.C.); vincenzo.ferrari@unipi.it (V.F.); 2 EndoCAS Center, Department of Translational Research and New Technologies in Medicine and Surgery, University of Pisa, 56126 Pisa, Italy; nadia.cattari@endocas.unipi.it (N.C.); roberta.piazza@endocas.unipi.it (R.P.); a.ruinato@studenti.unipi.it (A.D.R.); 3 Orthopaedic and Traumatology Division, Department of Translational Research and of New Surgical and Medical Technologies, University of Pisa, 56124 Pisa, Italy; simone.colangeli@ao-pisa.toscana.it (S.C.); paolo.parchi@unipi.it (P.D.P.); rodolfo.capanna@unipi.it (R.C.)

**Keywords:** mixed reality, surgical planning, surgical simulation, hybrid simulation, cryotherapy, Microsoft HoloLens

## Abstract

Cryosurgery is a technique of growing popularity involving tissue ablation under controlled freezing. Technological advancement of devices along with surgical technique improvements have turned cryosurgery from an experimental to an established option for treating several diseases. However, cryosurgery is still limited by inaccurate planning based primarily on 2D visualization of the patient’s preoperative images. Several works have been aimed at modelling cryoablation through heat transfer simulations; however, most software applications do not meet some key requirements for clinical routine use, such as high computational speed and user-friendliness. This work aims to develop an intuitive platform for anatomical understanding and pre-operative planning by integrating the information content of radiological images and cryoprobe specifications either in a 3D virtual environment (desktop application) or in a hybrid simulator, which exploits the potential of the 3D printing and augmented reality functionalities of Microsoft HoloLens. The proposed platform was preliminarily validated for the retrospective planning/simulation of two surgical cases. Results suggest that the platform is easy and quick to learn and could be used in clinical practice to improve anatomical understanding, to make surgical planning easier than the traditional method, and to strengthen the memorization of surgical planning.

## 1. Introduction

Cryoablation, also known as cryosurgery or cryotherapy, is a technique for ablating a target tissue and an appropriate surrounding margin through controlled freezing to lethal temperatures, without inducing damage to nearby vital organs [1].

Preceded by a near half-decade of investigations, modern cryosurgery dates back to 1961, when Cooper and Lee invented an automated cryosurgical system cooled by liquid nitrogen for treating Parkinsonism and other neural diseases [2]. Nowadays, preferred cryotherapy systems use the Joule–Thomson effect, taking advantage of the cooling of non-ideal gases when rapidly expanded. In these devices, a highly pressurized gas (e.g., argon) is expanded through a nozzle inside a small cylindrical steel sheath, and the Joule–Thomson expansion cools the sheath to cryogenic temperatures very quickly [3]. In clinical practice, single or multiple cryoprobes, that is, needle-shaped instruments, are inserted into the target structure and several freeze-thaw cycles are applied sequentially. As the argon flows through the needle, an ice ball forms around the tip of the cryoprobe, inducing tissue necrosis by direct mechanisms in the surrounding tissues. Moreover, in regions farther from the probe, cells are damaged as a result of several processes such as crystallization of water, cell dehydration, metabolic derangement and vascular stasis [4,5].

Nowadays, cryosurgery can be performed percutaneously or with an open surgery approach to treat a wide range of benign and malignant diseases including lung, breast, oesophagus, liver, kidney, prostate and bone cancers [6]. In orthopaedic oncology, cryoablation is well established for the palliative care of metastatic bone tumours, or as an adjuvant treatment. Indeed, bone tumours that are benign or have low metastatic potential can be treated by tissue-conserving techniques, commonly with intralesional excision by curettage followed by cryosurgery [7]. Applied after curettage, cryosurgery devitalizes an additional margin of tissue around the lesion, improving the effectiveness of the treatment, as suggested by cumulative data showing more than 90% recurrence-free outcomes with a 5-year average follow up [6]. Recently, our group published a retrospective study involving 143 patients in which cryotherapy proved its efficacy in further increasing the local control of disease compared to standard treatment without cryotherapy [8].

As with any surgical procedure, cryotherapy is expected to be most effective, in terms of accuracy and precision, when preceded by preoperative planning. Cryotherapy planning, however, is mostly performed qualitatively, based on the surgeon’s experience, and mostly after two-dimensional visualisation of radiological data sets. This can take several hours and the result can often be different from that expected [9]. Sub-optimal positioning of the cryoprobe leads to the following issues that jeopardize the quality of the surgical treatment and increase the intervention cost: incomplete freezing of the target lesion, inadvertent damage to surrounding healthy tissue, use of an unnecessarily large number of cryoprobes, increased duration of the surgical procedure, and increased likelihood of post-surgical complications [10]. The ideal planning tool, therefore, should allow the surgeon a clear understanding of the surgical anatomy and a simulation of cryoprobe insertion to ensure adequate coverage of the target volume while maximizing freezing damage to the target region and minimizing damage to the surrounding tissue [11]. More specifically, according to [1], surgeons need to be able to assess four critical variables for frozen mass formation, namely the type of the cryoprobe, the number of the cryoprobes, the configuration of the cryoprobes, and the duty cycle rate.

The importance of optimizing cryotherapy planning is widely described in the literature, and for several years computerized means have been proposed for modelling cryoablation through numerical heat transfer simulations [10,12,13,14,15,16]. For example, Baissalov et al. [14] describe a simulation algorithm, based on solving the transient heat conduction equation using finite element methods, to generate temperature distributions from cryoprobes, visualize isotherms in the anatomical region of interest, and provide tools for estimating damage to the target and surrounding structures. Lung et al. [10] present a computerized planning tool, which is more efficient than traditional numerical optimization techniques because it requires significantly fewer biological heat transfer simulations for each planning iteration. For demonstration purposes, 2D examples of typical cross-sections in prostate cryosurgery are provided. Keelan et al. [15] instead describe an efficient numerical technique for simulations on a graphics processing unit (GPU), to facilitate rapid decision making associated with cryosurgery training. A GPU-based simulation approach is also developed in [9,16] to achieve a high performance; indeed, for employing such planning tools in the daily clinical routine, patient-specific simulation of cryoablation needs to be not only sufficiently accurate but also fast. Moreover, the planning tool developed in [9], which uses a physics-based simulation, allows the user to interact with the application through gestures to be compatible with use in a sterile environment. Another example of GPU-based 3D ice ball modelling for cryoablation simulation and planning is the work presented in [17], the accuracy of which was validated on an ex-vivo warm gel setup and simulation on five retrospective patient cases of kidney tumour cryoablation. In [18], the authors developed a fast explicit dynamics finite element algorithm (FED-FEM) for the fast simulation and analysis of bio-heat transfer in soft tissue and studied a parallel implementation for real-time bio-heat computation. The system could be used for different thermal treatments but has not yet been tested for cryoablation procedures. Finally, in [19], a web-based planning tool for percutaneous cryoablation of renal tumours, based on a modified Pennes bioheat equation with an added perfusion term, is presented. However, validation results showed a tendency of the simulation model to overestimate the ablation effect, thus adjustments are necessary to make the tool suitable for clinical use. In conclusion, most software platforms are either still not accurate enough or do not meet some key requirements for routine clinical use, such as high computational speed and ease of use, or have not yet been tested in the specific area of cryotherapy.

This work does not focus on heat transfer modelling, but employs information about ice ball size and isotherms provided by the cryoprobe manufacturer (isotherms are derived after a 10-min cooling cycle in agar gel phantoms) as an input for the implementation of a platform for patient-specific planning and simulation of the optimal cryoprobe configuration (type, number, pose) to ensure complete ablation of a target lesion while avoiding damage to surrounding anatomical structures. More specifically, we aim to develop an intuitive platform that provides surgeons with a simple tool for anatomical understanding and pre-operative planning by integrating the information content of 3D radiological images and cryoprobe specifications either in a 3D virtual environment or in a hybrid scenario, which exploits the potential of 3D printing and augmented reality (AR). The main hypothesis of this work is that such a simulation platform, which allows both visual and tactile inspection of 3D anatomical models, can facilitate the understanding of the clinical case and ease/improve surgical planning. This hypothesis is based on the results of several studies that have demonstrated the value of 3D printing and AR for surgical planning and/or simulation.

For example, the value of 3D printing for understanding patient anatomy has been validated in [20] in the field of general surgery. According to this study, 3D printed models help transfer complex anatomical information to clinicians, proving useful for pre-operative planning, intra-operative navigation and surgical training purposes. Similar results were obtained in [21], which demonstrated that the use of 3D printing in orthopaedic surgical planning provides the surgeon with extra information that leads to a better surgery strategy, improves accuracy, and reduces procedure time, bleeding and the amount of anaesthesia.

As for AR technology, wearable systems based on head-mounted displays have the potential to shift the paradigm of how processed radiological datasets are commonly deployed in surgery, reducing the cognitive burden and improving information management in image-guided surgery. AR represents a particularly useful asset for improving the surgeon’s spatial perception of the surgical field, because it allows for the merging of the patient specific 3D models generated from preoperative images either contextually to the real patient’s anatomy, that is, as a tool for surgical navigation, or on a physical replica of the anatomy under treatment, that is, as a tool for surgical simulation/planning [22,23,24].

For these reasons, in [25,26], we proposed hybrid simulation platforms for training in orthopaedic procedures (i.e., pedicle screws fixation and hip arthroplasty, respectively) combining 3D printed patient-specific bone models with AR functionalities. More specifically, in [25], we performed qualitative tests to evaluate the workload and usability of the Microsoft HoloLens for our orthopaedic simulator, considering both visual and audio perception and interaction/ergonomics issues, and the results obtained encourage the use of the proposed hybrid approach for orthopaedic open surgery.

Based on these previous experiences, in this paper, we propose and test a simulation and planning platform for cryosurgery that consists of a standalone desktop application for the Microsoft Windows platform coupled with a hybrid simulation environment relying on 3D printed anatomical replicas enriched with HoloLens (version 1.0) mixed reality capabilities. To the best of the authors’ knowledge, this is the first work in the literature evaluating the use of AR and hybrid simulation for cryotherapy planning.

## 2. Materials and Methods

The following paragraphs describe the architecture of the desktop application and the hybrid simulation environment, and the protocol implemented for the preclinical qualitative tests.

### 2.1. Desktop Application

The software application, designed for a desktop computer (with Microsoft Windows 10 Operating System, 64-bit), integrates multiple modules/scripts to: visualize, explore, and measure the patient-specific 3D anatomical structures; and to simulate cryoprobe insertions to optimally plan their ideal number and proper placement according to the tumour location/extension. The application was developed on the cross-platform game engine Unity3D (Unity Technologies Inc., San Francisco, CA 94103, USA) (version 2019.3.15f1).

**Figure 1:**
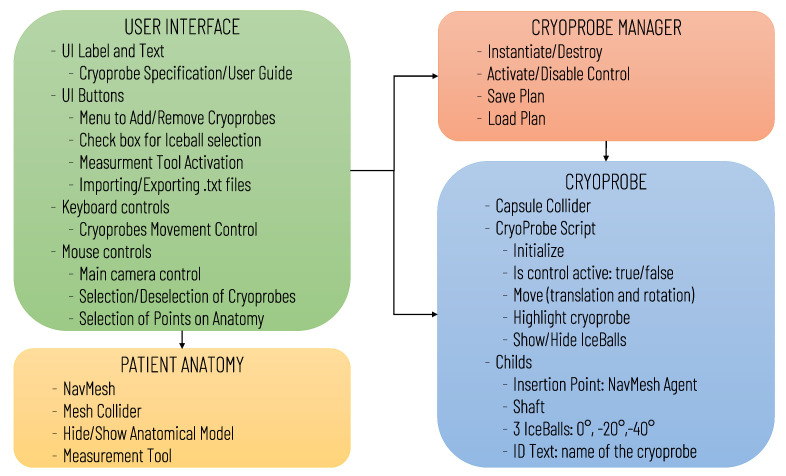
Diagram of the virtual simulator software architecture illustrating the main software modules and the data transfers between modules.

The software architecture, summarized in Figure 1, includes the following modules: User Interface, Patient Anatomy, Cryoprobe Manager, and Cryoprobe.

As for the Cryoprobe Module, a set of prefabs was developed according to the Cryoprobe producer specifications (Endocare inc., Irvine, CA, USA) for the simulation of the different available tools which differ in terms of the shape and size of the ice ball generated, and in the needle length and diameter. The ice ball sizes were derived from in-vitro studies using gelatin-based phantom, and, according to the producer, they approximate a performance (±5 mm) in soft tissue at 100% gas for 10 min; however, the actual isotherm may vary depending on patient-specific parameters [27].

**Figure 2:**
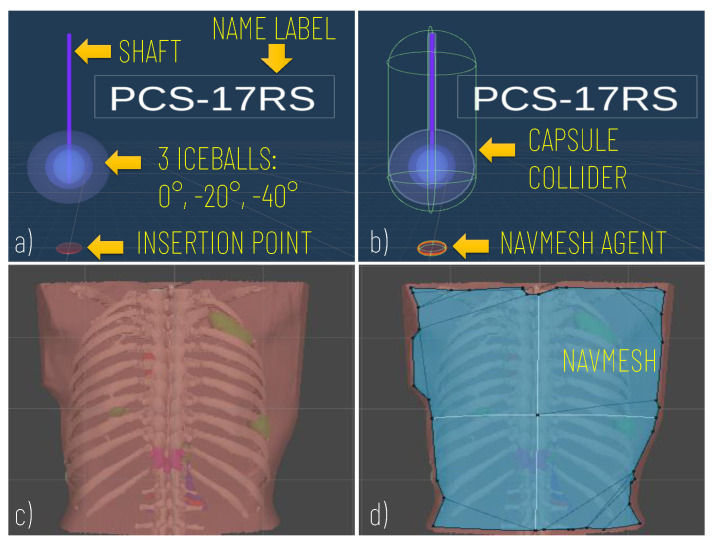
Modelling of the Cryoprobe and the anatomy. (**a**) A cryoprobe prefab comprising the model of the needle, three isotherm ice balls, the insertion point, and a label for the model’s name; (**b**) Unity components added to the cryoprobe prefabs: capsule collider to allow the cryoprobe selection via mouse click; (**c**) Example of the anatomical model imported; (**d**) The navigation mesh (in blue) baked on the torso of the patient and used to implement the interactive navigation of the insertion point on the skin).

Prefabs included the model of the Cryprobe needle, the three different isotherms (0°, −20°, −40°), a label for the Cryoprobe name, and a model of the insertion point to manage the control of instrument placement (Figure 2a).

**Figure 3:**
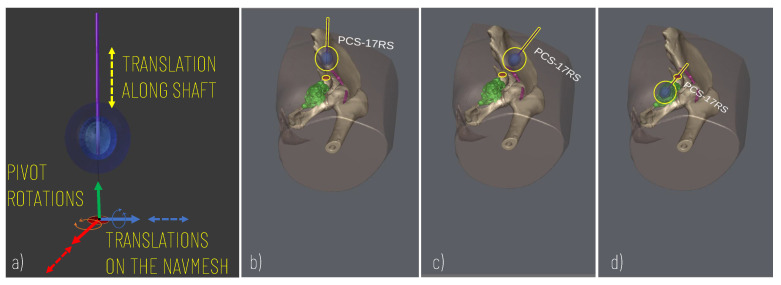
Simulation of the cryoprobe placement. (**a**) 5 DOF modelled for defining the best insertion point (2 DOF translations for displacement on the skin model), insertion axis (2 pivot rotations), and insertion depth (1 DOF translation). (**b**–**d**) Example of sequences of simulator scenes after pivot rotation (**c**) and translation (**d**) along the cryoprobe longitudinal axis.

To control the cryoprobe positioning to allow for accurate planning, the following degrees of freedom (DOF) are modelled (Figure 3): two translational DOF to manage the positioning of the cryoprobe insertion point along the patient’s body (the insertion point is constrained to move on the patient’s body and therefore two degrees of freedom entirely define its position on the body surface); two rotational DOF to manage the rotations of the needle around the insertion point and thus define the optimal orientation of the cryoprobe (given the axial symmetry of the needle and the ice ball, 2 DOF define the orientation); one additional translational DOF to move the cryprobe along its main axis and thus assess the optimal insertion depth.

The movement of the cryoprobe insertion point is implemented by exploiting a Unity NavMesh; this latter is a 3D navigation mesh approximating the “walkable” surface for a specific NavMesh Agent type. A NavMesh is generated on the skin model (Patient Anatomy Module) (Figure 2d) and properly configured to allow the movements of the cryoprobe insertion point, which is equipped with a NavMesh Agent component (Figure 2b).

The Cryoprobe Manager Module, based on user input, performs the following basic actions: it creates new instances of the cryoprobe prefab, destroys prefabs, activates/deactivates the control of each cryoprobe in the scene, saves the surgeon’s planning in a .txt file, and loads the result of previous planning. This latter includes, for each planned cryoprobe, the following information: model name, selected operating temperature, insertion point position in the local coordinate system of the anatomy (which coincides with the global reference system of the scene), and cryoprobe pose in the insertion point’s coordinate system (depicted in Figure 3a). At each time, only one of the instanced cryoprobe prefabs in the scene is “active” (i.e., the user can control its placement and select the different ice balls): the active probe is by default the last one inserted; however, the user can decide to switch to the control of another probe by selecting it with a simple click of the mouse (for the detection of this event each prefab is equipped with a collider as depicted in Figure 2b).

**Figure 4:**
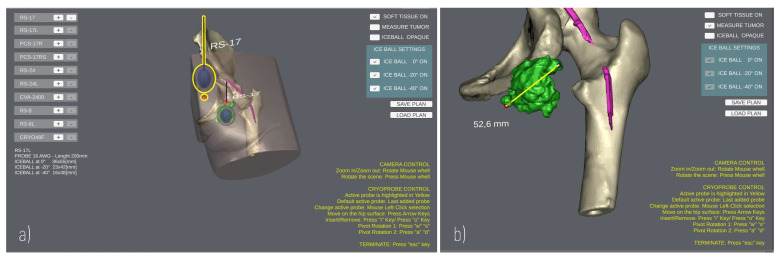
GUI during a planning. (**a**) Planning the positioning of two Cryoprobe. The “active” Cryoprobe is highlighted in yellow; (**b**) Example of measurement performed to evaluate the size of a tumour.

The User Interface enables the surgeon to add/remove cryoprobe models in the scene, and to rehearse the cryoprobe placement (Figure 4) using the mouse and a minimal set of keyboard keys. The translational degrees of freedom of the cryoprobe are managed by pressing the “WAS” and “I/” keys, which enable the movement of the cryoprobe insertion point on the patient’s skin and the translation of the cryoprobe needle along its longitudinal axis, respectively. The pivot rotations around the cryoprobe insertion point are managed via the arrow keys to determine the best insertion direction. A virtual trackball (implemented via a C# script component attached to the main camera) allows the user to zoom and rotate the main view using mouse buttons to explore the patient’s anatomy.

The Patient Anatomy Module includes all the anatomical models relevant for the surgical planning/simulation. The 3D virtual models are extracted from the available preoperative radiological dataset (CT or MRI) of the patient: the DICOM dataset is processed with a semi-automatic tool, the EndoCAS Segmentation Pipeline [28] is integrated into the open source software ITK-SNAP 1.5 [29], then the mesh reconstruction and optimisation steps are performed to generate the 3D models of the patient’s anatomy. The optimisation steps, including artefact removal, hole filling, mesh decimation and smoothing, are performed using the open-source software MeshLab [30] and Blender [31]. The latter is also used to set the anatomy local reference system at a convenient position for the next simulation phase with the HoloLens, as detailed in the next section.

Three-dimensional (3D) anatomical models are imported in the project, aligning their coordinate system with the scene global reference system; then, a NavMesh component is generated on top of the skin, and a mesh collider is added to the models so that the user can click points on the mesh surface for measurement purposes. Indeed, the Patient Anatomy Module includes a C# script to detect clicks on the anatomy and calculate the Euclidean distance between two clicked points (Figure 4b), obtaining the 3D coordinates of the impact points where rays, emitted by a Raycast, hit the collider.

### 2.2. Hybrid Simulator

**Figure 5:**
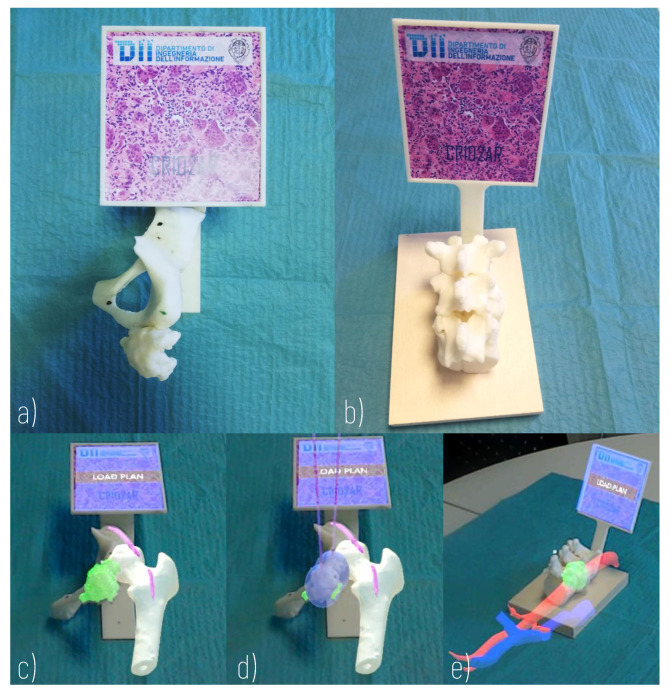
Hybrid Simulator. (**a**) Physical component of a simulator of arteriovenous malformation of the thigh (Surgical Case 1); (**b**) Physical component of a simulator of metastasis of the body of the D11 vertebra (Surgical Case 2); (**c**) AR view of Surgical Case 1; (**d**) AR view of Surgical Case 1 with loaded plan; (**e**) AR view of Surgical Case 2.

The hybrid simulation environment, which was developed with an approach similar to that described in [25], includes physical and virtual components (Figure 5).

The physical components include 3D printed anatomical replicas and a marker, rigidly anchored to the anatomical components, allowing the registration of the virtual content to the real scene. For this purpose, a support for the registration marker is designed according to the morphology of the anatomical replica and ergonomic evaluations concerning the user’s viewing direction. A 3D printer (Dimension Elite 3D Printer, with a building volume of 203 × 203 × 305 mm, and a maximum resolution of 0.178 mm) is used to turn the 3D CAD models into tangible 3D synthetic replicas made of acrylonitrile butadiene styrene (ABS). This plastic is commonly used for the manufacturing of bone replicas for orthopaedic surgery simulation, since it quite realistically replicates the mechanical behaviour of the natural tissue [32]. The anatomical parts to be printed are selected each time according to the specific surgical case: the design of the simulator requires the selection of the anatomical parts to be manipulated (those that can provide haptic feedback useful for the comprehensive understanding of the surgical case) and the parts that can be simply visualised in AR.

The virtual components, shown in AR thanks to the Microsoft HoloLens headset (version 1.0), include not only anatomical parts but also models of the cryoprobes, positioned according to the surgical planning performed with the virtual application, and loaded at runtime. Display-eye calibration and virtual-to-real registration should be performed to guarantee the spatial coherence of the virtual components and the 3D printed parts. As for the Display-eye calibration, the Microsoft HoloLens 1 official “Calibration” app is used, even if it does not offer a complete user-based calibration procedure (it is designed to solely adapt the two displays’ rendering engine to the user’s interpupillary distance [33]).

Virtual-to-real registration is achieved by exploiting:

- The HoloLens spatial localization, relying on the on-board optical and inertial sensors and on the proprietary Simultaneous Localization and Mapping (SLAM) self-tracking algorithm [34];
- Inside-out optical tracking of a target object, achieved using the functionalities offered by Vuforia SDK [35], and using a planar marker rigidly anchored to the 3D printed replica.

More specifically, a custom image target, which provides sufficient detail to be detected by the Vuforia Engine, is created with the Vuforia Target Manager using a .JPG image. The features extracted from the image are stored in a database, which can then be integrated into the software application and used by the Vuforia Engine for runtime image processing, allowing detection of a physical marker and the estimation of its pose in the HoloLens spatial mapping coordinate space. Once the Image Target is detected, the Vuforia Engine tracks whether it is at least partially visible from the HoloLens world-facing camera, otherwise the HoloLens’ spatial mapping and positional tracking systems are used to keep the virtual-to-real registration as stable as possible from any perspective [36].

From the software standpoint, Unity3D is used to develop the HoloLens application by exploiting the functionalities of the MixedRealityToolkit (MRTK), which is a collection of C# scripts and Unity components to develop mixed-reality applications targeting Windows Mixed Reality. The MRTK allows the user to interact with the virtual content through head movements, gestures and voice. A virtual cursor, the position of which is controlled by head movements (HoloLens v.1 does not include any eye-tracking sensor), is added to the application to interact with the user interface.

**Figure 6:**
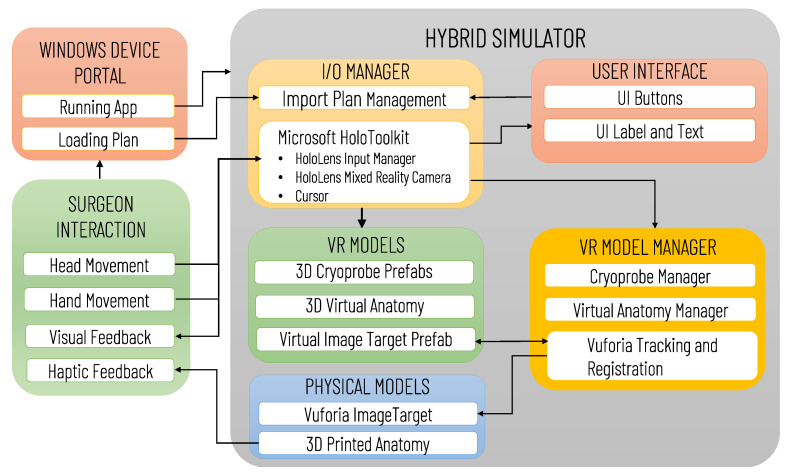
Architecture of the Mixed Reality Simulation Environment developed for Microsoft HoloLens. The diagram illustrates the main software (IO/Manager, User Interface, VR Models, VR Manager) and hardware (Physical Models) modules, the data transfers between modules, and the possible user (surgeon) interactions.

Figure 6 illustrates the architecture of the Mixed Reality Simulation Environment, highlighting the data transfers between software and hardware modules (e.g., 3D printed Anatomy and Vuforia Image Target), and the possible user interactions. The software modules comprise an I/O Manager, the User interface, VR Models and the VR Model Manager. The I/O Manager is based on the use of MRTK to handle the HoloLens mixed reality functionalities, allowing the user an intuitive interaction with the virtual content, and the importing of the preoperative plan via the Windows Device Portal (WDP). WDP is a web server that enables communication with the HoloLens Headset via the network connection, the management of the HoloLens apps, and the navigation/loading of files. The surgeon can load/reload the preoperative plan at any time, and can visualize in AR the last planned configuration/positioning of the cryoprobe models by clicking a UI button.

The VR model manager handles the control of the VR Models Module, which includes the 3D models of the anatomy, the cryoprobe prefabs and the Vuforia Image Target prefab, whose pose is updated in real-time according to the Vuforia Tracking and Registration Functionalities. The anatomy, as well as each cryoprobe prefab, are instantiated as a child of the Vuforia Image Target so that their Transform component is expressed in the Vuforia Image Target’s local reference system. For simplicity, the anatomy model is exported into the reference system of the Image Target, and the cryoprobe poses in the .txt plan file are expressed in the same system: thus, once the Vuforia marker pose has been determined, the virtual content can be easily aligned to the printed anatomical parts, without any further transformation.

**Figure 7:**
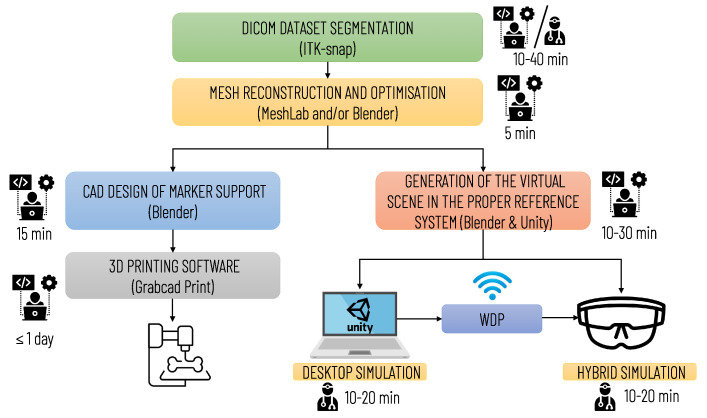
Diagram illustrating the operational steps for implementing the entire simulation and planning platform. The preparation of the simulation requires qualified technical personnel; the segmentation phase can be performed by medical personnel (by a radiologist or the surgeon himself/herself) or by technical personnel. In the latter case, the final approval of a radiologist is required.

Figure 7 summarizes the main steps for implementing the simulation, with an indication of the professional profiles (surgical staff and technical staff) involved in each step and the estimated time required. The time for implementing the simulation may vary depending on the quality of the diagnostic images and the number of anatomical structures to be included in the simulation. The duration of planning and simulation may vary depending on the surgeon’s level of experience and the complexity of the surgical case.

### 2.3. Experimental Evaluation

A qualitative study was carried out to preliminarily evaluate the proposed platform through the retrospective planning/simulation of the cryotherapy treatment of two surgical cases selected among patients operated on in the last year at the Cisanello Hospital (Pisa, Italy).

The first case was a 20-year-old woman with arteriovenous malformation (AVM) of the thigh, previously treated with multiple surgeries and embolizations (see Figure 3b–d, Figure 4, and Figure 5a,c,d). The cryotherapy procedure was performed under CT guidance and, given the proximity of the sciatic nerve to the lesion, evoked potentials were acquired initially and after each cryo-cycle for intraoperative monitoring. This case was selected to evaluate whether the proposed platform can help with the planning of cryoprobe insertion to completely ablate the target lesion while maintaining a safe margin from the nerve.

The second case was a 45-year-old male affected by metastatic renal cell carcinoma with metastasis of the body of the D11 vertebra (see Figure 2 and Figure 5b,e). The surgery involved a wide laminectomy, exposure of the tumoral mass, cryotherapy to cut down the bleeding of the tumour (indeed renal cell metastases are well known for their high and extended vascularity), and finally the debulking of the tumoral mass. This case was selected to test whether the proposed platform helps with planning the insertion of the probes into a lesion and to simulate their ideal pose while avoiding unintentional damage to the dural sac, and also to the aorta and vena cava.

Seven subjects, including orthopaedic surgeons and orthopaedic residents recruited from Cisanello Hospital, were enrolled as voluntary participants after giving written informed consent. Table 1 shows participants’ demographic information, including the level of experience in cryotherapy treatment, AR, Microsoft HoloLens and 3D printing.

A questionnaire collecting quantitative and qualitative (free text) data was administered to participants.The questionnaire comprised five closed *yes/no* questions, six open questions, and 29 Likert items, structured in six sub-questionnaires (named *Traditional Plan*, *Virtual Plan*, *Feedback on Virtual Plan*, *Hybrid Plan*, *Feedback on Hybrid Plan*, and *Feedback on the Entire Platform*) to be administered in successive phases. The sub-questionnaires were designed to progressively test the desktop application and the hybrid simulator, based on their planned sequence of use (desktop application followed by the hybrid simulation).

The study protocol and a description of the sub-questionnaires are presented below.

The protocol of the study included the following 13 steps:

1. Administration of the informed consent and demographic form (step 1);
2. Surgical planning based on the CT report, 2D visualisation and study of radiological images using a dicom viewer and cryoprobe specifications (including information on needle length and diameter, size of ice balls) (step 2);
3. Administration of the *Traditional Plan* sub-questionnaire (step 3);
4. Five minute explanation of desktop application functionality and GUI buttons, keyboard and mouse controls, followed by 5 min practice on a demo surgical case (step 4);
5. Surgical simulation and planning of a selected surgical case using the Desktop Application (step 5);
6. Administration of the *Virtual Plan* sub-questionnaire (step 6);
7. Administration of the *Feedback on Virtual Plan* sub-questionnaire (step 7);
8. Visualisation and manipulation of the 3D printed anatomical replica (step 8);
9. AR visualisation of the virtual plane (output of step 5) contextually blended with the 3D printed replica via Microsoft HoloLens (step 9);
10. Iterative repetition of steps 5 and 9 until the surgeon was satisfied with the surgical plan (step 10);
11. Administration of the *Hybrid Plan* sub-questionnaire (step 11);
12. Administration of the *Feedback on Hybrid Plan* sub-questionnaire (step 12);
13. Administration of the *Feedback on the Entire Platform* sub-questionnaire (step 13).

Participants were divided into two groups; each of the two groups was assigned one of the two surgical cases, and the other was used as a demo case for the training described in step 4. More specifically, the first group, which included two experts (18 and 12 years of experience in orthopaedic surgery) and two residents (2nd and 4th year of residency), was assigned case 1; whereas the second group, which included two experts (17 and 9 years of experience in orthopaedic surgery) and 1 resident (3rd year of residency) was assigned case 2.

The first sub-questionnaire, *Traditional Plan* (see Table 2), included two closed *yes/no* questions and two open questions aimed at assessing the ability of surgeons and residents to plan the number, type, and positioning of cryoprobes, based on: the CT report, 2D visualization and study of radiological images via a Dicom viewer, and cryoprobe specifications.

The second sub-questionnaire—the *Virtual Plan*—which included the same questions as the first part (see Table 2), was administered to assess the ability of the surgeons and residents to plan the surgery based on the desktop application.

The third sub-questionnaire—the *Feedback on Virtual Plan*—aimed to collect the subjects’ opinions regarding the ergonomics, functionality, and usefulness of the desktop application via 11 Likert items (in the Results Section) and included an optional open question to collect their suggestions for additional functionalities to be added (i.e., Describe the functionalities to be added to the Desktop application—optional to be completed only if a value lower than three has been assigned to item 5).

The fourth sub-questionnaire—the *Hybrid Plan*—consisted of a single closed *yes/no* question to assess whether the hybrid simulation leads to a re-evaluation/optimization of the surgical plan made with the desktop application (i.e., After using the hybrid simulator do you think the previous desktop planning should be modified? *yes/no*).

The fifth sub-questionnaire—the *Feedback on Hybrid Plan*—aimed to collect the subjects’ opinions about the ergonomics, functionality, and usefulness of the desktop application through 15 Likert items (in the Results Section), and an optional open question to collect their suggestions for potential features to be added (i.e., Describe the functionalities to be added to the Hybrid application—optional to be completed only if a value lower than three has been assigned to item 6).

Finally, the sixth sub-questionnaire, named *Feedback on the Entire Platform*, consisted of three Likert items (in the Results Section) to collect the subjects’ opinions about the potentialities of the complete platform (simulation/planning through the desktop application followed by the hybrid simulation) to increase the safety of surgery, facilitate the placement of cryoprobes, and reduce the time of surgery.

## 3. Results

All participants, regardless of their level of experience in cryotherapy, claimed to be able to plan the intervention (probes type, number and positioning) with the traditional approach (*Traditional Plan* questionnaire).

Table 3 summarizes the plan modifications after desktop and hybrid planning. Three out of seven participants preferred to modify their original plan after using the desktop application (*Virtual Plan* questionnaire): more specifically, three out of four participants, that is, two residents and one expert from the first group (surgical case 1) modified their plan, whereas no participants from the second group (surgical case 2) modified their original plan. Finally, three out of seven participants refined their plan after the hybrid simulation (*Hybrid Plan* questionnaire): one resident from the first group further modified his virtual plan, and two participants, that is, one expert and one resident from the second group, modified their original plan for the first time.

Table 4 summarizes participants’ feedback on the Desktop Application, collected by the administration of the *Feedback on Virtual Plan* questionnaire. The results collected are positive for both ergonomics and usability aspects as well as perceived utility: overall, participants agreed or strongly agreed with each statement, except for item 5 “I would not add more functionality to the desktop application”, and item 8 “The desktop application is useful for a clear understanding of the spatial relationship between the lesion and adjacent anatomical structures,” for which a neutral opinion was expressed. Regarding this last item, two subjects expressed a neutral opinion and two subjects a negative one (disagree). It should be noted that three of these four participants expressed a positive opinion concerning the statement “The hybrid application is useful for a clear understanding of the spatial relationship between the lesion and adjacent anatomical structures” (item 9 of the *Feedback on Hybrid Plan* questionnaire). One participant, on the other hand, also expressed a neutral opinion regarding the hybrid application and, in both cases, suggested enriching the simulation with the visualization of more anatomical structures (e.g., muscles and vessels). Regarding item 5, four participants suggested adding the following features:

1. Replacement of the probe without changing the plane (insertion point, insertion depth, and direction) (suggested by one resident);
2. Visualization of the probe insertion axis (suggested by one resident);
3. Selection of anatomical elements to make transparent (suggested by one trainee);
4. Calculation of anatomical structures volume (suggested by one resident);
5. Visualization of more anatomical structures (suggested by one trainee and one senior surgeon);
6. Two-dimensional (2D) visualization of the surgical plan superimposed on CT images (suggested by one expert).

Table 5 summarizes participants’ feedback on the Hybrid Simulator, collected by the administration of the *Feedback on Hybrid Plan* questionnaire. In this case, the results collected were also positive for both ergonomics and usability aspects as well as perceived utility: overall, participants agreed or strongly agreed with each statement, except for item 4, “I believe the hybrid application provides all the essential information for preoperative planning”, item 5 “The hybrid application offers a more ergonomic view of the anatomy and surgical plan than the desktop application” and item 7 “I would like to do the whole simulation and planning with the hybrid application (without using the desktop application)” for which a neutral opinion was expressed; furthermore participants disagreed with item 6 “I would not add other functionalities to the hybrid application”. As for perceived usefulness, participants agreed that the hybrid application is useful for the clear assessment of lesion margins (item 8), the spatial relationship between the lesion and adjacent anatomical structures (item 9), and the distances between the lesion and adjacent anatomical structures (item 10). Furthermore, according to the participants, the hybrid simulator allows for a better memorization of the surgical plan compared to classical planning (item 11), and a better memorization and understanding of the anatomy compared to desktop planning (item 12 and item 13); and the tactile interaction with the printed models helps with the understanding and memorization of the anatomy (item 14). Moreover, all participants expressed strongly positive feedback about the use of the hybrid simulator as a didactic tool, based on a library of real surgical cases, for the training of novices in cryotherapy (item 15).

Regarding item 6, four participants suggested:

1. The addition of physical replica of muscles and skin (suggested by two trainees and two experts);
2. The addition of interactions with virtual cryoprobes to modify their pose without the use of the Desktop application (suggested by one expert);
3. The addition of respiration motion simulation (suggested by one expert);
4. The improvement of AR registration (suggested by one trainee and one expert);
5. The addition of measurement tool (suggested by one trainee).

Table 6 summarizes participants’ feedback on the entire platform. Participants agreed or strongly agreed with all three items, supporting that the proposed technologies can increase the safety of the intervention (item 1), facilitate the positioning of the cryoprobes (item 2) and also have the potential to shorten intervention times (item 3).

## 4. Discussion

The introduction of technologically advanced and affordable cryotherapy devices, along with the results of medical studies suggesting the efficacy and benefits of cryotherapy and cryosurgery for the treatment of a wide variety of diseases, have boosted the popularity of these techniques in recent years. Despite its increasing adoption in several surgical specialties, cryosurgery today is mostly planned qualitatively, based on the surgeon’s experience, and mostly after two-dimensional visualization of radiological data sets [9]. Since the 1990s, a significant amount of scientific work has focused on modelling cryoablation through numerical simulations of heat transfer; however, one of the major limitations of adopting these simulations in clinical practice is the computational time, which can be massively high [17]. Today, probe manufacturers make information available on cryoprobe performance by evaluating ice ball size and temperature penetration in phantom gel models. Although these models are not complete, for example, they do not provide information on the impact of heat input from the surrounding tissue or on the ablative zone created [37], they provide essential information for surgeons for selecting the type and number of probes necessary to treat the lesion/s.

In this work, we present and preliminarily test a platform aimed at improving anatomical understanding and pre-operative planning through the integration of a 3D model of the patient’s anatomy, extracted from preoperative radiological images, and cryoprobe specifications, either in a 3D virtual environment or in a hybrid scenario, which exploits the potential of 3D printing and AR.

The developed platform has been tested for retrospective simulation of two surgical cases and the results obtained are very encouraging, although they need to be confirmed by a study with a larger number of surgeons. The combined use of the desktop application and the hybrid simulator allowed surgeons to refine their cryotherapy plan in terms of probe number, type and pose: not only the residents, but also some of the more experienced surgeons, modified their original plan and elaborated upon traditional information (CT scan visualization, report and cryoprobe specifications) after the use of the desktop application and/or the hybrid simulator.

All participants confirmed that the use of the desktop application and the hybrid simulator is easy and quick to learn and that both tools can be used in clinical practice. Moreover, the feedback on the utility of the two systems is positive: both allowed a clear evaluation of the lesion margins and its distance from the adjacent anatomical structures, making the surgical planning easier than with the traditional method. Based on the results obtained, the efficiency of the simulation was enhanced with the use of the hybrid application, which is a valid tool for use in conjunction with the desktop application since it improves the understanding of the spatial relationships of the involved anatomical structures, and it strengthens the memorization of surgical planning through visualization and manipulation of full-scale anatomical models.

Finally, according to this preliminary preclinical study, surgeons believe the proposed platform has the potential to increase surgical safety, reduce operative time, and facilitate cryoprobe placement in the operating room. To validate these preliminary results, a prospective, randomized, single-center clinical trial is now being conducted to compare the short- and long-term outcomes of cryotherapy procedures performed with traditional planning with those planned and simulated with our platform.

According to suggestions received by the surgeons recruited in the study, the number of anatomical structures involved in each simulated case will be increased (e.g., including muscles if the quality of the DICOM dataset allows for their segmentation), and software refinements will be performed (e.g., to allow volume calculation).

Our platform, to date, is limited by the lack of patient-specific modelling of temperature evolution and phase changes in the tissue, as we use static ice ball models based on information provided by the manufacturer, derived from in-vitro studies on gelatin-based phantoms. In the future, we plan to integrate such modelling within the desktop application and allow the simulation of the duty cycle rate. One strategy could be to perform an initial simulation of the cryoprobe placement based on static models (as today) and then refine the simulation with heat diffusion modelling, considering the dependence of tissue parameters on their temperature.

## Figures and Tables

**Table 1:** Demographics of participants in the user study.

General Info	Value
Profession/Position Held (orthop. residents; orthop. surgeons)	(3; 4)
Gender (male; female; non-binary)	(7; 0; 0)
Age (min; max; mean; STD)	(28; 43; 35; 6)
Experience (in years) in Orthopaedic Surgery (min, max, mean, STD)	(2; 18; 9; 7)
Experience (in years) in Cryotherapy (min, max, mean, STD)	(1; 10; 4; 3)
Experience with Augmented Reality (none, limited, familiar, experienced) *	(3; 3; 1; 0)
Experience with Microsoft HoloLens (none, limited, familiar, experienced) *	(5; 1; 1; 0)
Experience with 3D printing (none, limited, familiar, experienced) *	(1; 2; 4; 0)

* none = technology never used; limited = technology used less than once a month; familiar = technology used about once a month; experienced = technology used several times a month. STD = Standard Deviation; AR = Augmented Reality.

**Table 2:** Traditional/Virtual Plan Questionarrie.

Question	Answer
Based on * are you able to: Plan the type of probes?	Yes/No
If yes, indicate the type of probes.	——
Based on * are you able to: plan the probe number and positioning?	Yes/No
If yes, indicate the number and describe the plan.	——

* Traditional Plan Questionnaire Option—Based on the information traditionally available for planning (CT, CT report, cryoprobe specifications); Virtual Plan Questionnaire Option—Based on the Desktop application.

**Table 3:** Plan modifications after desktop and hybrid planning.

Surgical Case	Position Held	After Desktop Application	After Hybrid Simulation
1	Resident	Probe pose	Probe pose
1	Resident	Probe type	No changes
1	Surgeon	Addition of one probe	No changes
1	Surgeon	No changes	No changes
2	Resident	No changes	Probe type
2	Surgeon	No changes	Probe orientation
3	Surgeon	No changes	No changes

**Table 4:** Five point Likert questionnaire results for the Feedback on the Virtual Plan (from 1 = strongly disagree, to 5 = strongly agree). The central tendency of responses is presented by the median, with the dispersion presented by the interquartile range (iqr).

		Item	Median	iqr
Ergonomics & Usability	1	The desktop application is intuitive to operate	4	(4–5)
	2	Learning to use the desktop application takes little time	5	(4.5–5)
	3	I believe the desktop application can be used in clinical practice	5	(5–5)
	4	I believe the desktop application provides all the essential information for preoperative planning	4	(2.5–4.5)
	5	I would not add more functionalities to the desktop application (if you disagree with this statement, please suggest which functionality should be added)	3	(2–3.5)
	6	I would use this app even on its own (without 3D printing)	4	(2.5–4.5)
Utility	7	The desktop application is useful for clearly assessing the margins of the lesion	5	(4.5–5)
	8	The desktop application is useful for a clear understanding of the spatial relationship between the lesion and adjacent anatomical structures	3	(2.5–4.5)
	9	The desktop application is useful for clearly assessing the distances between the lesion and adjacent anatomical structures	4	(3.5–4)
	10	The desktop application makes it easier to plan the type of probes than traditional planning	5	(4–5)
	11	The desktop application makes it easier to plan the positioning and number of probes than traditional planning	4	(4–4.5)

**Table 5:** Five point Likert questionnaire results for the Feedback on the Hybrid Plan (from 1 = strongly disagree, to 5 = strongly agree). The central tendency of responses is presented by the median, with the dispersion presented by the interquartile range (iqr).

		Item	Median	iqr
Ergonomics & Usability	1	The hybrid application is intuitive to operate	5	(4–5)
	2	Learning to use the hybrid application takes little time	4	(4–5)
	3	I believe the hybrid application can be used in clinical practice	4	(4–5)
	4	I believe the hybrid application provides all the essential information for preoperative planning	3	(2–3.5)
	5	The hybrid application offers a more ergonomic view of the anatomy and surgical plan than the desktop application	3	(2–5)
	6	I would not add other functionalities to the hybrid application (if you disagree with this statement please suggest the functionalities to be added)	2	(2–2.5)
	7	I would like to do the whole simulation and planning with the hybrid application (without using the desktop application)	3	(2–3.5)
Utility	8	The hybrid application is useful for clear assessment of lesion margins	5	(4–5)
	9	The hybrid application is useful for a clear understanding of the spatial relationship between the lesion and the adjacent anatomical structures	4	(3.5–4)
	10	The hybrid application is useful for clearly assessing the distances between the lesion and adjacent anatomical structures	4	(3–4.5)
	11	The hybrid application allows a better memorization of the surgical planning than the classical planning	5	(4–5)
	12	Hybrid application allows better memorization of the surgical planning than the desktop application	4	(3–4.5)
	13	3D printing is useful for a better understanding of anatomy than the virtual desktop application	4	(4–4)
	14	Being able to touch the printed model helps me understand the anatomy and memorize it	4	(4–4.5)
	15	The hybrid application can be used for cryotherapy training by novices using a library of already treated surgical cases	5	(5–5)

**Table 6:** Five point Likert questionnaire results for the Feedback on the Entire Platform (from 1 = strongly disagree, to 5 = strongly agree). The central tendency of responses is presented by the median, with the dispersion presented by the interquartile range (iqr).

	Item	Median	iqr
1	The proposed new planning and simulation tools make it possible to increase the safety of the operation	4	(4–5)
2	The new planning and simulation tools facilitate the positioning of cryoprobes in the operating theatre	4	(4–5)
3	The proposed new planning and simulation tools allow for shorter intervention times	5	(4–5)

## Data Availability

The data presented in this study are available on request from the corresponding author.

## Abbreviations

The following abbreviations are used in this manuscript:

AR	Augmented Reality
ABS	Acrylonitrile Butadiene Styrene
CAD	Computer Aided Design
CT	Computed Tomography
DOF	Degrees Of Freedom
GPU	Graphics Processing Unit
I/O	Input/Output
MRTK	MixedRealityToolkit
SDK	Software Development Kit
SLAM	Simultaneous Localization And Mapping
VR	Virtual Reality
WDP	Windows Device Portal
